# The Root Causes of the Limited Electroluminescence Stability of Solution-Coated Versus Vacuum-Deposited Small-Molecule OLEDs: A Mini-Review

**DOI:** 10.3389/fchem.2022.857551

**Published:** 2022-04-08

**Authors:** Fatemeh Samaeifar, Hany Aziz

**Affiliations:** Department of Electrical and Computer Engineering and Waterloo Institute for Nanotechnology, University of Waterloo, Waterloo, ON, Canada

**Keywords:** extrinsic, intrinsic, OLEDs, solution-coating, stability, vacuum-deposition

## Abstract

Using solution-coating methods for the fabrication of organic light-emitting devices (OLEDs) offers a tremendous opportunity for enabling low-cost products and new applications. The electroluminescence (EL) stability of solution-coated (SOL) OLEDs, however, is significantly lower than that of vacuum-deposited (VAC) OLEDs, causing their operational lifetimes to be much shorter—an issue that continues to hamper their commercialization. The root causes of the lower EL stability of these devices remain unclear. This article briefly reviews and summarizes some of the work that has been done to-date for elucidating the root cause of the lower EL stability of SOL OLEDs, giving special attention to studies where side-by-side comparisons of SOL and VAC devices of the same materials have been conducted. Such comparisons allow for more-reliable conclusions about the specific effects of the solution-coating process on device stability to be made. The mini-review is intended to introduce the work done to-date on the causes of lower stability in SOL OLEDs and to stimulate further work for the purpose of closing the existing knowledge gap in this area and surmounting this long-standing challenge in the SOL OLED technology.

## Introduction

Organic light-emitting devices (OLEDs) are increasingly being used in commercial flat display products from mobile phones and smart watches to televisions (Reineke, 2015). Although OLEDs have become a recognizable product to consumers only recently, their exceptional potential over competing display technologies—liquid crystal displays (LCDs) primarily—has been demonstrated for decades (Reineke, 2015; Shin et al., 2016). While LCDs use back lighting, OLEDs are self-emissive, making it possible for each pixel to be turned on or off individually, resulting in lower power draw and deeper black levels (Reineke, 2015; Shin et al., 2016; Chen et al., 2018). Perhaps one of the most unique properties of OLEDs arises from their low-temperature fabrication process, which allows for the use of flexible plastic substrates and thus inexpensive large-scale processing (Shin et al., 2016). Further down the line, the possibility of fabricating OLEDs via solution-coating processes presents an opportunity for lower-cost applications, especially solid-state lighting products (Reineke, 2015).

There have been several phases in OLED development, from the first demonstration of a fluorescent material-based device by Tang and Van Slyke (1987) to the much more efficient phosphorescent material-based devices used in today’s commercial products. Recently, the use of thermally activated delayed fluorescence (TADF) materials, which can offer comparable efficiencies to phosphorescent materials without the use of heavy metals, is emerging as a third generation in this technology (Tao et al., 2014). Throughout these developments, the ability to realize OLEDs with sufficiently high electroluminescence (EL) stability has always lagged behind the progress in efficiency (Kondakov et al., 2007).

From a fabrication standpoint, OLEDs can be made via one of two approaches: vacuum-deposition or solution-coating. In vacuum-deposition, the various layers of an OLED (each typically a few 10 s of nms in thickness at most) are formed by thermal evaporation of the materials (usually in a powder or granular form initially) in a low pressure environment to produce a thin film of the material on the substrate. Production-scale vacuum-deposition systems come in two basic configurations: cluster, where the chambers are around a central substrate-handling robot, and in-line, where the chambers are in a line and the substrate moves from one end to the other. Both cluster and in-line approaches are currently used in the manufacturing of commercial OLED products since they allow for making complicated multiple-layer devices and give excellent device performance (Tang and VanSlyke, 1987; Burroughes et al., 1990; D'Andrade and Forrest, 2004; Forrest, 2004; Shirota, 2005; Tao et al., 2008; Reineke et al., 2009; Jou et al., 2010; Kamtekar et al., 2010; Ye et al., 2010; Zhang et al., 2010; Cai et al., 2011; Coya et al., 2012; Wang et al., 2012; Zhang et al., 2012; Chang and Lu, 2013; Fu et al., 2013; Liu et al., 2014; Ho et al., 2015; Liu et al., 2015; Liu et al., 2019). Vacuum-deposition-based approaches, however, have major drawbacks such as high equipment cost, high vacuum requirements, and complicated color patterning processes (Shirota, 2005; Kim et al., 2007). Cluster systems also suffer from inefficient utilization of materials. Solution-coating, in contrast, involves forming the layers via a wet-coating method (e.g., spin coating, blade coating, or inkjet printing) after dissolving the materials in a suitable organic solvent, capitalizing on the uniqueness of organic materials to offer soluble electroluminescent materials. These techniques, especially inkjet printing and blade coating, provide significant advantages in terms of material utilization and fabrication costs, especially for large-area products (Forrest, 2004; Duggal et al., 2007; Hou et al., 2008; Kim et al., 2008; Duan et al., 2010; Hou et al., 2010; Singh et al., 2010; Zhong et al., 2011; Minakata et al., 2016). Inkjet printing also allows for easy color patterning, offering additional advantages in reducing fabrication costs (Klauk, 2006).

Early generations of solution-coated (SOL) OLEDs were made primarily using polymeric semiconductors, which, in comparison to conjugated small-molecule materials, are generally easier to dissolve (Burroughes et al., 1990). However, difficulties with obtaining very high purities, narrow molecular weight polydispersity, and batch-to-batch reproducibility in polymers have spurred an interest in developing soluble small-molecule materials (Duan et al., 2010; Yook and Lee, 2014). Because of the significant progress in this front over the last decade, small-molecule OLEDs with very impressive efficiencies, made by solution-coating, are now possible (Jou et al., 2010; Yook and Lee, 2011; Lee et al., 2013; Yook and Lee, 2014; Cho et al., 2015; Han et al., 2016). Despite this progress, the EL stability of SOL OLEDs continues to be significantly lower in comparison with their vacuum-deposited (VAC) counterparts (Lee et al., 2009; Duan et al., 2010; Cai et al., 2011; Yook and Lee, 2011; Zhang et al., 2012; Cho et al., 2016; Han et al., 2016; Cho et al., 2017; Stolz et al., 2017). The short lifetime is currently the main obstacle preventing the commercialization of low-cost OLEDs via solution-coating.

In general, failure in OLEDs is caused by various degradation mechanisms that can be classified into two categories: ambient-induced degradation and electrical stress-induced degradation (Kondakov et al., 2007; Sudheendran Swayamprabha et al., 2021). Ambient-induced degradation appears in the formation of localized defects, induced by various ambient-driven reactions, that lead to the growth of non-emissive areas in the device (i.e., dark spots) over time and often also leading to electrical shorts (Burrows et al., 1994; Burrows and Forrest, 1994). Electrical-stress-induced degradation, in contrast, appears in the form of a gradual decrease in the internal quantum efficiency (IQE) of the devices, without any visible defects. The behavior is caused by various physical and chemical changes that take place in the materials under electrical stress, induced by the flow of charges or by the resulting excitons. These changes can be influenced by factors including the device architecture and fabrication process (Rothberg and Lovinger, 1996; Aziz et al., 1999; Matsumura et al., 1999; Kondakov et al., 2003; Kondakov, 2005; Kondakov et al., 2007; Luo et al., 2007; Giebink et al., 2008; Giebink et al., 2009; Wang and Aziz, 2013; Wang et al., 2014; Wang and Aziz, 2015a; Wang and Aziz, 2015b; Zhang and Aziz, 2016; Dong et al., 2017). Although degradation mechanisms in OLEDs have been extensively studied and are relatively well-understood, the root causes of the lower stability of SOL OLEDs relative to their VAC counterparts remain largely unclear at this time. Closing this knowledge gap is critical for successfully surmounting the poor stability challenge of SOL OLEDs and propelling the technology toward commercialization.

**FIGURE 1:**
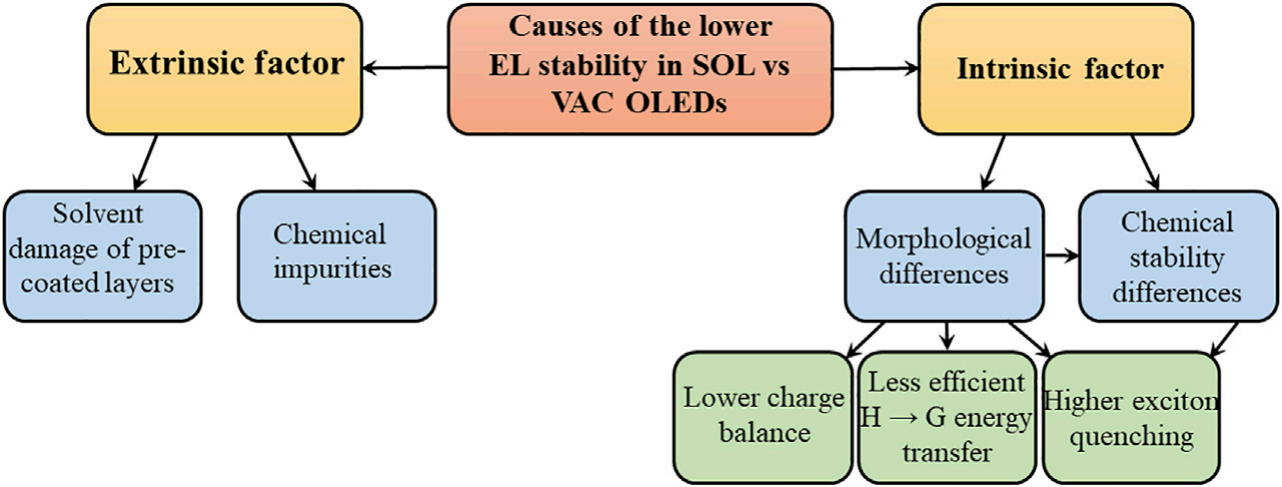
Extrinsic and intrinsic factors behind the lower EL stability in SOL versus VAC OLEDs.

Several factors uniquely affect SOL OLEDs and may contribute to their lower stability. These factors can generally be divided into extrinsic and intrinsic. Extrinsic factors, broadly defined as external to the specific material(s) or layer(s) being coated by the solution-coating process, include 1) the negative effects of the solvents used in the layer(s) being coated on other device layers, especially pre-coated ones, and 2) the unintentional introduction of impurities into the device from either the solvents or the coating environment. Intrinsic factors, in contrast, can be defined as inherent to the nature of the layer(s) made by solution-coating such as specific characteristics in their 1) morphologies and 2) chemical reactivity, both of which may lead to lower device stability. Figure 1 maps out the extrinsic and intrinsic factors behind the lower EL stability in SOL versus VAC OLEDs.

This article briefly reviews and summarizes some of the work that has been done to-date directed at elucidating the root causes of the shorter operational lifetime of SOL OLEDs, giving special attention to studies that perform side-by-side comparisons of SOL versus VAC devices made of the same materials, and thus allow for more-reliable conclusions about the specific effects of the solution-coating process on device stability to be made. The first part reviews some of the work that has focused on understanding and addressing extrinsic factors and covers solvent damage of pre-coated layers and chemical impurities, whereas the second part covers some of the investigations into the intrinsic factors, covering the morphological and chemical stability differences between the SOL and VAC OLEDs. The mini-review is intended to serve as an introduction to efforts so far on addressing the question of the causes of the lower stability of SOL OLEDs and to stimulate further work for the purpose of closing the existing knowledge gap in this area.

## Extrinsic Factors

### Solvent Damage of the Pre-coated Layers

Unlike in the case of vacuum-deposition, where the coating of one layer does not significantly disturb other precoated layers, solution-coating involves coating a layer with a liquid solution of the material in a solvent, which makes it easier to disturb or alter the other layers that have been precoated on the asubstrate. The solvent can penetrate into the underlying layers causing swelling and/or morphological changes in them, or even their partial (or complete) removal. Significant mixing along the boundary between the layers can also occur. Such changes to the underlying layers would obviously negatively affect the device performance and contribute to lower stability as was reported previously (Lin et al., 2015; Lan et al., 2019; Zheng et al., 2020).

One approach to avoid or mitigate the effects of solvent damage to underlying layers is to use chemically cross-linkable materials in these layers, utilizing thermal or photochemical cross-linkers (Gather et al., 2007; Rehmann et al., 2007; Huang et al., 2008; Köhnen et al., 2009; Liaptsis et al., 2013; Liaptsis and Meerholz, 2013; Wang et al., 2020). Using cross-linkable 4.4′, 4″-tris-(N-carbazolyl)-triphenlyamine (TCTA) derivatives, Liu et al. showed that the present efficiency of devices with a SOL-hole transport layer (HTL) could be preserved (Liu et al., 2008). A similar observation was reported by Niu et al., (Niu et al., 2007). While photo cross-linking is generally more efficient and can be used for creating more robust layers more quickly, its reliance on the use of photoacids makes it inevitable for a residual amount of side products or initiators to remain in the final layer, which might impair the device efficiency and stability. Thermal cross-linking is therefore considered to be a better option, especially for device stability (Ho et al., 2015; Chao et al., 2019). Using PLEXCORE^®^, a thermally cross-linked HTL, Xiang et al. demonstrated OLEDs with efficiency and stability comparable with those with a VAC 4,4′-bis [N-(1-naphthyl)-N-phenylamino]biphenyl (NPB)-based HTL (Xiang et al., 2014). The light emission layer (EML) of these devices was, however, made by vacuum-deposition in both cases. A similar effect was observed in devices with thermally cross-linked 9,9-bis{4-[(4-ethenylphenyl)methoxy]phenyl}-N2,N7-di-1-naphthalenyl-N2,N7-diphenyl-9H-fluorene-2,7-diamine (VB-FNPD) HTLs, where exposing the layers to solvents was found to have no detrimental effect on device efficiency or stability (Samaeifar et al., 2021).

Another approach for avoiding solvent damage to the underlying layers is to use, in the different layers, materials that are only soluble in orthogonal solvents (Gong et al., 2005; Ye et al., 2011; Aizawa et al., 2014; Wang et al., 2020). The search for suitable pairs of solvents can be aided by determining their Hansen solubility parameters (Hansen, 2007). However, recent work has shown that even nonsolvents (i.e., solvents that can dissolve only insignificant amounts of the layers underneath) could still change the surface of the substrate on which the layers were coated and affected interlayer interfaces (Fujii et al., 2009; Yu and Aziz, 2021). In fact, other work has shown that even in cases where the underlying layers remained intact, exposure to solvents may still alter their surface properties such as work function, sheet resistance or roughness (Wang Q et al., 2011; Wang et al., 2013; Yun et al., 2016). While the exact implications of these phenomena on the performance of SOL OLEDs remain to be worked out, one could expect changes at the interlayer interfaces to affect charge injection or transport, and, as a result, charge balance (Aziz et al., 1999; Xia et al., 2007; Davidson-Hall and Aziz, 2020; Yu and Aziz, 2021). Changes in charge balance can lead to charge accumulation at various device interfaces such as the HTL/EML and the electron transport layer (ETL)/EML interfaces, where they can facilitate non-radiative processes. (Kim et al., 2007; Lee et al., 2009; Reineke et al., 2013).

### Chemical Impurities

By virtue of their nature, the low-pressure environments used in vacuum-deposition naturally allow for a better avoidance of unintentional contaminants that may get introduced into the materials during OLED fabrication and negatively affect the efficiency and stability (Fujimoto et al., 2016; Kaur et al., 2016; Fujimoto et al., 2020). Also, many OLED materials are sensitive to oxygen and moisture, and although inert gas environments can be used to provide some protection, the lower-pressure environments of the alternative are more effective for avoiding ambient species (Aziz and Popovic, 2004; Baldacchini et al., 2005; Knox et al., 2006). High-vacuum environments also help minimize the particle contamination, caused by the movement of gases in the solution-coating environment during device fabrication (Gotthold, 2015). Such particle contamination, which is more common in solution-coating processes, can induce undesired pathways for current leakage, causing device efficiency to deteriorate over time (Gather et al., 2007; Oostra, 2016). Such leakage currents also facilitate the formation of microscopic shorts in the devices when under electrical stress, resulting in hot spots, possibly leading to catastrophic device failure (Oostra et al., 2014). Controlling the impurity and particle levels in the solution-coating environment is therefore critical for improving SOL OLEDs’ performance and stability. Another source of impurities in SOL OLEDs could be the solvents (Gaspar and Polikarpov, 2015). Therefore, special attention needs to be paid not only to the solubility and purity of the OLED materials (Becker et al., 2010; Duan et al., 2010), but also to the purity of the solvents used for solution-coating. Liu et al. revealed that impurities in the solvents used in preparing the SOL EMLs may have a leading role in the short operational lifetime of these devices. Therefore, in order to fabricate SOL OLEDs with longer operational lifetimes, solvents with ultra-high purity levels are necessary (Liu et al., 2016).

## Intrinsic Factors

### Morphological Factors

Several studies have investigated the differences between small-molecule SOL and VAC films in regards to density, molecular orientation, surface roughness, and glass transition temperature. Kim et al. reported that SOL films of the N,N′-Di(1-naphthyl)-N,N′-diphenyl-(1,1′-biphenyl)-4,4′-diamine (NPB):2,2′,2"-(1,3,5-benzenetriyl)-tris [1-phenyl-1H-benzimidazole] (TPBi):tris(1-phenylisoquinoline)iridium (Irpiq)_3_ composite had a lower refractive index than their VAC counterparts, despite having similar average roughness values. The lower refractive index was attributed to the lower molecular packing density in the former, which was also believed to be the cause of the higher driving voltage of these devices (Kim et al., 2007). Similarly, Lee et al. showed that the densities of 2-(t-butyl)-9,10-bis(20-naphthyl)anthracene (TBADN) doped with 4,4′-bis{2-[4-(N,N-diphenylamino)phenyl]vinyl} (DPAVBi) films processed from toluene and chlorobenzene solutions were much lower than those of VAC films of the same materials. They also observed that the root-mean square roughness values of SOL films were quite similar to those of VAC films (Lee et al., 2009). We have shown that the VAC film and SOL film using toluene exhibited similar surface topography, whereas films using dichloromethane and chloroform exhibited higher roughness values (Cho and Aziz, 2018). Xing et al. showed that VAC TCTA films had a highly oriented molecular arrangement with face-to-face π−π stacking, whereas SOL films had a much more random molecular morphology (Xing et al., 2013). In a systematic study covering a large number of small molecular materials, Shibata et al. found that the film density, glass transition temperature, and degree of horizontal molecular orientation were lower in SOL films than the corresponding VAC ones. They also showed that the glass transition temperature and molecular orientation of SOL films of glassy materials were identical to those of “deteriorated” VAC films that had experienced a transition induced by heating (Shibata et al., 2015).

The lower glass transition temperature of SOL systems can be expected to directly lead to a lower thermal and temporal morphological stability in these systems (Tokito et al., 1997). Naturally, any morphological changes that occur in device layers after fabrication can negatively affect the device performance, as they would lead to structural defects and non-homogeneities in charge transport that can, in turn, accelerate degradation processes. We may, therefore, conclude that the lower EL stability of devices made by solution-coating may—at least in part—be due to reduced morphological stability in these systems (Shirota and Kageyama, 2007).

Aside from the lower glass transition temperature of SOL systems, which would reduce their structural stability, differences in film density, molecular orientation, and surface roughness can also be expected to affect the intermolecular charge transport and energy transfer in these systems (Yokoyama, 2011), in turn, also affecting device stability (Luo et al., 2007; Giebink et al., 2009). While SOL TCTA films were shown to have a lower hole transport mobility compared with their VAC counterparts (Xing et al., 2013), an opposite effect was observed in SOL N,N′-bis(3-methylphenyl)-N,N′-diphenyl-(1,1′-biphenyl)-4,4′-diamine (TPD) films. OLEDs utilizing a SOL TPD layer as an HTL showed significantly higher currents and luminance levels at any given voltage relative to devices with a VAC TPD layer (Feng et al., 2011). A similar observation was reported by Ishihara et al. in OLEDs with SOL TPD or N,N′-di (p-biphenyl-4-yl)-N,N′-diphenyl-(1,1′-biphenyl)-4,4′-diamine (p-BPD) HTLs (Ishihara et al., 2004). Despite some variations in the observations (Mao et al., 2011; Wang et al., 2011a; Wang et al., 2011b; Lee and Lee, 2013; Mangalore et al., 2019; Kuznetsov et al., 2021), there is broad agreement that SOL and VAC films exhibit significant differences in their charge transport characteristics.

Liu et al. investigated the degradation mechanisms in small-molecule phosphorescent OLEDs with SOL versus VAC EMLs and found that SOL EML devices were more prone to hole-induced degradation, especially in the presence of excitons. They also found that the degradation rate in SOL devices depended on the initial hole injection/transport properties. Follow-up studies revealed that good hole injection and transport properties were required in SOL OLEDs to suppress interfacial degradation (Liu et al., 2016). However, Lee et al. suggested that using materials with enhanced hole-blocking and electron-transporting properties was essential for improving the efficiency and stability in SOL EML devices in order to offset the higher hole mobility and electron trapping characteristics in these layers relative to VAC systems (Lee et al., 2009).

We have studied degradation mechanisms in single organic-layer devices and found that SOL layers had more charge traps. With a higher concentration of charge traps, exciton–polaron interactions and exciton quenching by polarons become more efficient, which accelerates the deterioration in device EL output. SOL layers were also found to exhibit more significant electromer formation. The increased formation of electromers points to increased morphological and structural defects in these films relative to those in their VAC counterparts, possibly arising from non-homogeneities in the extent of intermolecular interactions and/or molecular packing density from one location to another within the film (Cho et al., 2017).

While differences in the initial morphology in SOL versus VAC films are believed to play a role in the lower stability of the former, other studies have shown that SOL films may also be more susceptible to aggregation while under electrical stress, driven primarily by exciton–polaron interactions (Cho et al., 2016). The phenomenon is facilitated by the relatively lower molecular packing density and larger free volume in SOL films, both of can which allow for less-restricted molecular reorganization and mobility.

**FIGURE 2:**
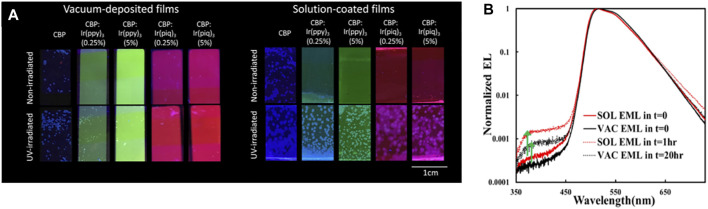
**(A)** Fluorescence microscopy images of neat and guest-doped CBP films subjected to UV irradiation for 18 h and of non-irradiated control films. 2,2′,2″-(1,3,5-benzinetriyl)tris(1-phe-nyl-1H-benzimidazole) [Ir (ppy)_3_] and tris(1-phenylisoquinoline)iridium [Ir (piq)_3_] used as guests. All films were thermally annealed at 100°C for 10 min to enhance crystallization. Reprinted with permission from Yu and Aziz (2020). Copyright 2020 American Chemical Society. **(B)** EL spectra (normalized to the guest emission peak intensity) collected initially (i.e., at t = 0) and after reaching the LT50 point of SOL or VAC EML devices. Reprinted with permission from Samaeifar et al. (2021). Copyright 2021 American Chemical Society.

A recent work has shown that exciton stress leads to larger losses in PL quantum yield and in host-to-guest (H → G) energy transfer in host: guest (H: G) EML systems fabricated by solution-coating. In a well-dispersed H: G system, most host molecules will be located within a few angstroms of a guest molecule and can therefore transfer energy efficiently to the guest, which reduces the exciton concertation in the host. Solution-coating produces film morphologies with some initial phase separation into guest-rich and guest-deficient domains, and the host/guest aggregation accelerates the formation of guest-deficient domains. As a result, it is more difficult for excited host molecules in these domains to lose their excitation energy to guest molecules as quickly, in turn, making them more susceptible to exciton-induced degradation and aggregation (Samaeifar and Aziz, 2022). Photoluminescence images showing the increased H: G phase separation and aggregation in SOL versus VAC 4,4′-bis-(carbazol-9-yl)biphenyl (CBP) films doped with various phosphorescent guests are reproduced in Figure 2A (Yu and Aziz, 2020). As can be seen, SOL films show much more extensive crystalline features after UV-irradiation. The higher susceptibility to crystallization indicates that SOL films have host/guest aggregates initially, which can act as nucleation sites for host/guest crystallization. The findings revealed the influence of the initial film morphologies produced by the different fabrication methods on energy transfer and material stability under exciton stress.

More recently, we found that the less efficient H → G energy transfer in H:G systems can also be detected in their EL characteristics (some of those observations are reproduced in Figure 2B). As seen, the relative intensity of the host emission band (here CBP at around 400 nm) increases after electrical driving in both devices. The increase is, however, larger in the case of the SOL EML device, despite the shorter electrical stress time. Since the detection of host emission points to incomplete H → G energy transfer, the observations suggest that energy transfer was initially somewhat less efficient in the case of SOL EML. This phenomenon was found to play a direct role in the lower stability of phosphorescent OLEDs based on H:G systems made by solution-coating (Samaeifar and Aziz, 2022). The observations again pointed to differences in molecular distribution or morphology in the case of SOL layers, with more H:G phase separation compared with their VAC counterparts. Solubility limitations could be playing a role in this increased H:G phase separation, resulting in the less-efficient H → G energy transfer in these systems (Samaeifar et al., 2021).

While a significant amount of effort has been devoted to studying and comparing the morphology in SOL versus VAC films and its effect on the differences in charge transport and exciton dynamics between these systems, it should be noted that, in many cases, the findings are material- and process-dependent (Mao et al., 2011; Wang et al., 2011a; Wang et al., 2011b; Lee and Lee, 2013; Mangalore et al., 2019; Kuznetsov et al., 2021). For example, although several studies reported that SOL films have lower density and glass transition temperature, Feng et al. observed that SOL TPD films were more compact and had a higher density than their VAC counterparts (Feng et al., 2011). Similarly, Kuznetsov et al. found that the density of tta: 2-thenoyltrifluoroacetone, DPPZ: dipyrido(3,2-a:2′c,3′c-c)phenazine [Eu (tta)_3_DPPZ]: CBP films made by solution-coating was higher than that obtained with vacuum-deposition (Kuznetsov et al., 2021). These observations show that, while there may be some morphological commonalities between SOL films, the strong dependence of the morphology on the specific materials and process conditions makes it difficult to generalize phenomena observed in one material system to another without direct verification.

### Chemical Stability Factors

While morphology plays a significant role in the lower stability of SOL OLEDs, chemical stability may also be another contributory factor. It should also be pointed out that differences in morphology may themselves lead to differences in chemical stability (Ito et al., 2012; Ahmadi and Wu, 2019; Geng et al., 2020). For example, in their studies of various solar-cell polymers, Mateker et al. observed a clear correlation between polymer-packing density and its photo-stability. They also showed that the rate of degradation becomes slower upon increasing film density, and that, regardless of the choice of the materials, films with crystalline morphology generally exhibited a higher photo-stability (Mateker et al., 2015). One may therefore similarly expect the lower molecular-packing density in SOL films to reduce their chemical stability.

We investigated and compared SOL and VAC OLED films under prolonged excitation using UV irradiation to determine if the lower lifetime of SOL devices is primarily due to the aggregation in the films or if chemical degradation also contributed to this effect. The results showed that the SOL film had more UV-induced chemical by-products formed under the irradiation conditions, indicating that chemical decomposition was faster relative to the VAC counterpart. Interestingly, the lower stability of SOL films was not due to any new (additional) chemical reactions or decomposition routes that occur in SOL films, suggesting that the faster chemical decomposition of SOL films by the UV irradiation has its origins in the different morphological make-up of these systems which makes the molecules less chemically stable relative to those in their VAC counterparts. We also found that the degradation rate also depends on the choice of solvents used in the solution-coating process (Cho and Aziz, 2018). The changes in the stability with the type of solvent is due to the formation of different polymorphs (Gleason et al., 2014). More work must be done in this area to better understand the effect of chemical stability on the shorter lifetime of SOL devices versus their VAC counterparts.

## Conclusion

In summary, we have reviewed some of the work to-date on elucidating the root causes of the lower EL stability of SOL OLEDs relative to their VAC counterparts, addressing certain factors at play. These factors can generally be classified into extrinsic and intrinsic ones. The former involves factors that are *external* to the material(s) or layer(s) being coated, such as contamination by impurities or solvent-damage effects. The intrinsic factors, in contrast, involve phenomena that are *inherent* to the nature of the layer(s) produced by solution-coating such as differences in their morphologies or chemical stability that in turn negatively affect the device stability. While the extrinsic factors can generally be controlled via corrective measures, our understanding of the intrinsic factors seems to be more elusive. Among the intrinsic factors, morphology seems to play a major role as it affects several factors that directly affect stability, such as charge transport (and therefore charge balance), H → G energy transfer, and degradation by excitons. However, other intrinsic factors, especially the question of reduced chemical stability, need to be investigated further. This mini-review is intended to serve as an introduction to work done to-date on addressing the causes of the lower stability of SOL OLEDs and to stimulate further work directed at closing the existing knowledge gap in this area and surmounting this long-standing challenge in SOL OLED technology.

## Data Availability

The original contributions presented in the study are included in the article/Supplementary Material, further inquiries can be directed to the corresponding author.
